# Prevalence and comorbidity of autism spectrum disorder in Spain: study protocol for a systematic review and meta-analysis of observational studies

**DOI:** 10.1186/s13643-019-1061-1

**Published:** 2019-06-14

**Authors:** Ferrán Catalá-López, Manuel Ridao, Isabel Hurtado, Amparo Núñez-Beltrán, Ricard Gènova-Maleras, Adolfo Alonso-Arroyo, Aurelio Tobías, Rafael Aleixandre-Benavent, Miguel A. Catalá, Rafael Tabarés-Seisdedos

**Affiliations:** 1 Department of Health Planning and Economics, National School of Public Health, Institute of Health Carlos III, Madrid, Spain; 2 Department of Medicine, University of Valencia/INCLIVA Health Research Institute and CIBERSAM, Valencia, Spain; 3 Instituto Aragonés de Ciencias de la Salud (IACS), Red de Investigación en Servicios de Salud en Enfermedades Crónicas (REDISSEC), Zaragoza, Spain; 4 Fundación para el Fomento de la Investigación Sanitaria y Biomédica de la Comunitat Valenciana (FISABIO-Salud Pública), Red de Investigación en Servicios de Salud en Enfermedades Crónicas (REDISSEC), Valencia, Spain; 5 Independent Clinical Psychologist and Researcher, Madrid, Spain; 6 Directorate General for Public Health, Madrid Regional Health Council, Madrid, Spain; 7 Department of History of Science and Documentation, University of Valencia, Valencia, Spain; 8 Information and Social and Health Research Unit (UISYS), University of Valencia and Spanish National Research Council (CSIC), Valencia, Spain; 9 Spanish National Research Council (CSIC), Barcelona, Spain; 10 Institute for Innovation and Knowledge Management (INGENIO)/Spanish National Research Council (CSIC) and Polytechnic University of Valencia (UPV), Valencia, Spain; 11 Faculty of Medicine, University of Valencia, Valencia, Spain

**Keywords:** Autism spectrum disorder, Comorbidity, Epidemiological study, Meta-analysis, Prevalence, Spain, Systematic review

## Abstract

**Background:**

Autism spectrum disorder (ASD) is a complex developmental disorder characterised by impaired social interaction and communication, and restrictive and repetitive behaviour. Previous systematic reviews have traditionally assessed the prevalence of ASD on global or regional context, with very few meta-analyses at the country level. The objective of this study will be to systematically evaluate published and unpublished observational studies that present prevalence and comorbidity of ASD among children, adolescent and adult population in Spain.

**Methods/design:**

We designed and registered a study protocol for a systematic review and meta-analysis of descriptive epidemiology data. Observational studies (cohort, cross-sectional) reporting the prevalence of ASD and conducted in a wide range of people (e.g. general population, outpatient and/or school settings) will be included. The primary outcome will be the prevalence of ASD. Secondary outcomes will be the prevalence of any physical or mental comorbidity in association with ASD. No limitations will be imposed on publication status, study conduct period, and language of dissemination. Comprehensive literature searches will be conducted in seven electronic databases (from January 1980 onwards), including PubMed/MEDLINE, EMBASE, Scopus, Web of Science, PsycINFO, IME—Spanish Medical Index and IBECS—Spanish Bibliographic Index of Health Sciences. Grey literature will be identified through searching dissertation databases, Google Scholar and conference abstracts. Two team members will independently screen all citations, full-text articles, and abstract data. Potential conflicts will be resolved through discussion. The study methodological quality (or bias) will be appraised using an appropriate tool. If feasible, we will conduct random effects meta-analysis of observational data. Prevalence estimates will be stratified according to gender, age and geographical location. Additional analyses will be conducted to explore the potential sources of heterogeneity (e.g. methodological quality, sample size, diagnostic criteria).

**Discussion:**

This systematic review and meta-analysis of observational data will identify, evaluate and integrate the epidemiological knowledge underlying the prevalence of ASD in Spain. The results of this study will be of interest to multiple audiences including patients, their families, caregivers, healthcare professional, scientists and policy makers. Results will be published in a peer-reviewed journal. Implications for future epidemiological research will be discussed.

**Systematic review registration:**

PROSPERO CRD42018090372

**Electronic supplementary material:**

The online version of this article (10.1186/s13643-019-1061-1) contains supplementary material, which is available to authorized users.

## Background

Autism spectrum disorder (ASD) is a complex developmental disorder characterised by early-onset deficits in social communication and reciprocal interactions as well as the presence of restricted, stereotypical behaviour [1–4]. ASD presents a set of behavioural problems that may produce functional impairment in social, school, or work performance and in the everyday activities of patients and their families [1, 2, 5, 6]. Pathogenesis of the disorder is not completely understood, but there may be many different causes that make a child more likely to have an ASD, including genetic predisposition, environmental and psychosocial factors [1–4].

The prevalence of ASD is now considered to be around 1% in many countries and regions worldwide [7–11], although estimates of 2% have been suggested in some published reports [7, 12]. Similar prevalence estimates have been reported in adults [13, 14]. ASD seems to affect more male than female individuals [15], and comorbidity is common (e.g. more than 50% may present concurrent physical or mental conditions) [1, 16–21]. Most recent global burden of disease estimates revealed 62.2 million people with ASD around the world in 2016 [6].

Systematic reviews of epidemiological data (whether at a national, regional or global levels) are important in the description of the geographical distribution of health problems [22, 23]. Systematic reviews of epidemiological data can also identify gaps in knowledge and inform future health care planning and research agendas [24]. Specifically, they can be useful to get more precise estimates of disease frequency, monitor trends and changes in disease burden over time, and contribute to the design of further studies (e.g. etiological and interventional studies). Previous systematic reviews and meta-analyses have traditionally assessed the prevalence of ASD on global or regional contexts [7, 8, 25–28], with very few examples of descriptive epidemiology meta-analyses conducted at the country level (e.g. India, China, Hong Kong and Taiwan) [29–31].

In recent years, several observational studies have been conducted in different geographical locations and population groups [7]. According to recent epidemiological studies, in Spain, the prevalence of ASD would be 0.61% in children aged 18 months to 3 years in the Canary Islands [32], 0.85% in children aged 0–14 years in Galicia [33], 0.92% in children aged 18 months to 3 years in Castile-León [34], and 2% in samples of children aged 3–6 years in Catalonia [35]. To the best of our knowledge, no previous systematic reviews and meta-analyses have evaluated ASD prevalence data in Spain. Therefore, it would be relevant to identify, select, and critically appraise the relevant epidemiological literature on ASD and to collect and analyse data from studies through integrated and iterative approaches.

The objective of this study will be to systematically evaluate published and unpublished observational epidemiological studies that present prevalence and comorbidity of ASD among children, adolescent and adult population in a Southwestern European country: Spain.

## Methods

### Protocol

This study protocol is part of an ongoing evidence synthesis project on the descriptive epidemiology and surveillance of neurodevelopmental disorders [36]. The present protocol has been registered within the PROSPERO database (registration number CRD42018090372) and is being reported in accordance with the reporting guidance provided in the Preferred Reporting Items for Systematic Reviews and Meta-Analyses Protocols (PRISMA-P) statement [37, 38] (see checklist in Additional file 1).

### Information source and literature search

The primary source of literature will be a structured search of seven major electronic databases (from January 1980 onwards—considering the *Diagnostic and Statistical Manual of Mental Disorders*, Third Edition [DSM-III] was published in 1980): PubMed/MEDLINE, EMBASE, Scopus, Web of Science, PsycINFO, and also national databases including IME—*Índice Médico Español* [Spanish Medical Index] and IBECS–*Índice Bibliográfico Español en Ciencias de la Salud* [Spanish Bibliographic Index of Health Sciences]). The secondary source of potentially relevant material will be a search of the grey or difficult to locate literature, including two dissertation databases (TESEO—*Base de datos de Tesis Doctorales* [Spanish Data Base of Doctoral Thesis Dissertations] and ProQuest Dissertations and Theses Database), Google Scholar and conference abstracts from selected national or local symposia on mental health, neurology and paediatrics. We will perform hand-searching of the reference lists of included studies, relevant reviews, national clinical practice guidelines or other relevant documents. Content experts and authors who are prolific in the field will be contacted. The literature searches will be designed and conducted by the review team which includes two experienced health information specialists. Our main literature search will be peer-reviewed by a senior health information specialist using the Peer Review of Electronic Search Strategies (PRESS) checklist [39]. The search will include a broad range of terms and keywords related to autism, epidemiological studies and the geographical area ‘Spain’. For the section of geographic area, the search will be based on a previously validated filter to minimise potential bias regarding the indexing of geographical items [40]. This filter is constructed around three complementary approaches: (1) the term ‘Spain’ and its variants in various languages, (2) terms related mainly to region and province place names, and (3) acronyms for regional health services. A draft search strategy for PubMed/MEDLINE is provided in Additional file 2.

### Eligibility criteria

Studies will be selected according to the following criteria: participants, condition or outcome(s) of interest, study design and context.

- Participants (population): We will include studies involving children, adolescents and adult population (regardless of age or sex).
- Condition or outcome(s) of interest: The primary outcome will be the prevalence of ASD indicating the number of people that have the disorder divided by the population number at a given point in time. This is often presented as a (prevalence) proportion. In this study protocol, we use the term ‘autism spectrum disorder’ to refer to both autistic disorder (e.g. the ‘classic’ definition of autism) and ‘other autism spectrum disorder combined’ (e.g. Asperger’s disorder; pervasive developmental disorders not otherwise specified, including atypical autism; Rett’s disorder; and childhood disintegrative disorder) as recommended elsewhere [7]. We will use author-reported definitions (according to accepted diagnostic criteria, such as the Diagnostic and Statistical Manual of Mental Disorders (DSM) or the International Classification of Diseases (ICD) criteria: ICD-9: 299.00, 299.10, 299.80; ICD-10: F84). Secondary outcomes will be the prevalence of any comorbidity, indicating the existence of any distinct additional (physical or mental) condition in association with ASD (e.g. according to main DSM-IV or ICD-10 categories of diagnoses).
- Study design and context: Eligible studies will be observational studies (cohort, cross-sectional or health surveys) reporting prevalence data using validated or non-validated tools and conducted in a wide range of people in the Spanish general population, outpatient (including data from administrative databases and registries) and/or school settings. Cross-sectional studies will be the most appropriate study design to determine the prevalence of ASD. Cross-sectional health surveys are typically used to estimate the point prevalence of common conditions of long duration and are generally less frequently used for rare diseases such as ASD. For cohort studies, only the first phase (cross-sectional) data will be considered. We will exclude studies in hospital/inpatient clinical settings because they are likely to be highly selected resulting in inaccurate estimations of the ‘true prevalence’ of the disorder (selection bias).

No limitations will be imposed on publication status (unpublished studies will be eligible for inclusion), study conduct period, and language of publication.

### Screening and selection procedure

All articles identified from the literature search will be screened by two team members independently. First, titles and abstracts of articles returned from initial searches will be screened based on the eligibility criteria outlined above. Second, full texts will be examined in detail and screened for eligibility. Third, references of all considered articles will be hand-searched to identify any relevant report missed in the search strategy. Any disagreements will be resolved by discussion to meet a consensus, if necessary. A flow chart showing details of studies included and excluded at each stage of the study selection process will be provided [41].

### Data collection

A data extraction form will be designed and used to extract equivalent information from each study report. Information of interest will include the following:

- Study characteristics: study design, year of publication, journal, year (or period) of study conduct, sample size, setting (community, school or outpatient), geographical location of study conduct: North (Galicia, Asturias, Cantabria, Aragon, Basque Country, Navarre, La Rioja), Mediterranean (Balearics, Catalonia, Valencia), Centre (Castile-La Mancha, Castile-León, Madrid, Extremadura), and South-East (Andalusia, Murcia and Canary Islands); and other fields to capture data relevant to the assessment of study methodological quality (see risk of bias and methodological quality assessment subsection).
- Participant characteristics: population sampled, age (e.g. mean with standard deviation, range) and gender (e.g. percentage of female participants).
- Outcome results: definitions and assessment tools (e.g. DSM-III, DSM-IV, ICD-9, ICD-10, other), data collection method (e.g. self-complete, questionnaire, interview and examination, clinical records), prevalence estimates (e.g. number of subjects with the disorder, proportion and 95% confidence interval) and any prevalence estimates stratified by age, gender, severity or location. If outcome results (e.g. proportion and 95% confidence interval) are not directly provided and it is feasible, we will calculate them from the number of cases and sample size provided in each single study.

Data extraction forms will be piloted initially on a small number of included studies. Subsequently, each of the included studies will be abstracted by two team members, independently, and potential conflicts will be resolved through discussion. Authors of primary publications will be contacted for data clarifications or missing outcome data, as necessary.

### Risk of bias and methodological quality assessment

The risk of bias of primary observational studies will be evaluated using a methodological quality critical appraisal checklist proposed in the Joanna Briggs Institute (JBI) systematic review methods manual [42, 43]. This methodological quality checklist for observational studies reporting prevalence data considers: sample representativeness, recruitment appropriateness, sample size, description of subjects and setting, coverage of data analysis, ascertainment and measurement of the condition, thoroughness of reporting statistical analysis, and identification and accountability of potential confounding factors/subgroups (see Additional file 3). We will provide a narrative summary of the risk of bias of the included studies, which will be supported by a table showing the results of the critical appraisal. Stars or points will be awarded for each quality item, and the highest quality studies will be awarded up to ten stars. Studies will be judged to be at low risk of bias (≥ 7 points), moderate risk of bias (4–6 points) or high risk of bias (< 4 points). The risk of bias for each observational study will be independently assessed by two reviewers. Discrepant scores will be resolved by discussion and consensus.

### Methods for evidence synthesis

The data from each paper (e.g. study characteristics, context, participants, outcomes and findings) will be used to build evidence tables of an overall description of included studies. Crude prevalence estimates (number of cases/sample size) will be presented along with 95% confidence intervals.

If feasible and appropriate, prevalence data points from primary observational studies will be used to perform random effects meta-analyses. Since heterogeneity is expected a priori, we will estimate the pooled prevalence and its 95% confidence interval using the random effects model with logit transformation and back transformation as recommended elsewhere [8, 22]. The random effects model assumes the study prevalence estimates follow a normal distribution, considering both within-study and between-study variation. Forest plots will be used to visualise the extent of heterogeneity among studies. Prevalence estimates will be expressed as cases per 10,000 people [8, 31].

We will quantify statistical heterogeneity by estimating the variance between studies using *I*^2^ statistic [44]. The *I*^2^ is the proportion of variation in prevalence estimates that is due to genuine variation in prevalence rather than sampling (random) error. *I*^2^ ranges between 0% and 100% (with values of 0–25% and 75–100% taken to indicate low and considerable heterogeneity, respectively). We will also report Tau^2^ [45] and Cochran *Q* test [46] with a *P* value of < 0.05 considered statistically significant (heterogeneity).

### Additional analyses

If sufficient studies are identified and data points are available, potential sources of heterogeneity will be investigated further by subgroup or meta-regression analyses according to baseline characteristics and methodological covariates. We plan to conduct analyses by gender (male vs female), age (e.g. children vs adolescent vs adult, mid-point of age range as continuous variable), severity (e.g. mild, moderate or severe), geographical location (e.g. North, Mediterranean, Centre and South-East), setting (e.g. community/school vs outpatient), sample size (e.g. < 1000, 1000–5000 or > 5000 participants), decade of publication (e.g. 1990, 2000 or 2010), study quality (e.g. low/moderate vs high-risk of bias), autism definition (e.g. autistic disorder vs other autism disorders combined), diagnostic system (e.g. DSM vs ICD criteria) and most recent diagnostic criteria (e.g. ‘DSM-IV or ICD-10’ vs ‘Not DSM-IV or ICD-10’). In addition, we will explore prevalence trends with gender variations (in terms of the female-to-male prevalence ratios) [15] and over time (with the year of publication as the explanatory variable) [1] using random effects meta-regression models [47]. Small study effects will be assessed by inspection of the funnel plots for asymmetry and with Egger’s test [48] and Begg’s test [49], with the results considered to indicate potential small study effects when *P* values < 0.10.

### Software considerations

All analyses will be conducted in Stata version 15 (StataCorp LP, College Station, Texas, USA) [50, 51].

### Ethics, dissemination and research integrity

No ethical approval is required for the performance of this study. The proposed systematic review and meta-analysis will be reported in accordance with the reporting guidance provided in the Preferred Reporting Items for Systematic Reviews and Meta-analyses (PRISMA) statement [41] and the Meta-analysis Of Observational Studies in Epidemiology (MOOSE) reporting guideline [52]. Any amendments made to this protocol when conducting the study will be outlined and reported in the final manuscript. Results will be disseminated through conference presentations and publication in a peer-reviewed journal. All data underlying the findings reported in the final manuscript will be deposited in a cross-disciplinary public repository—for example, the Open Science Framework (https://osf.io/) or Zenodo (https://zenodo.org/).

### Patient and public involvement

One of the study protocol co-authors (AT) is the parent of a child with ASD and a member of a patient group (APRENEM Association for the Inclusion of People with ASD, Barcelona, Spain). We will evaluate whether the epidemiological studies included in the systematic review had any patient and/or public involvement [53, 54].

## Discussion

The systematic review and meta-analysis of observational data presented in this protocol will identify, collect, evaluate and integrate the epidemiological knowledge underlying the prevalence of ASD in Spain. We are not aware of another systematic review and meta-analysis addressing this specific issue. In our opinion, this systematic review will help to establish the extent of the epidemiological evidence on ASD at a country level, in a reproducible and rigorous way.

The results of this study will be of interest to multiple audiences (including patients, their families, caregivers, healthcare professionals, researchers, scientists and decision makers). For example, the National Plan for Autism was issued in 2015 to develop specific measures to improve quality of life, promote awareness and identify and respond to the needs of those living with ASD [55]. The National Plan considered several strategic lines in terms of diagnosis, integrated care, health care, education, employment and research, among others. However, routine population estimates of ASD prevalence (based on the most updated systematic literature reviews) are not reported [55, 56]. Outputs of this analysis could be relevant to current policy questions.

There are several strengths and limitations of our planned systematic review methods. We will comprehensively evaluate epidemiological data (both published and unpublished) characterising the prevalence and comorbidity of ASD, exploring the extent of heterogeneity and potential biases in observational studies. We anticipate that we will identify knowledge gaps to be filled by new epidemiological research considering that the prevalence of neurodevelopmental disorders has been poorly covered in the literature [7, 57]. On this regard, implications for future epidemiological research will be discussed in the final manuscript. A key challenge is that based on knowledge from previous reviews on child mental health [7, 8, 25, 31, 57–59], we anticipate identifying studies using different study designs, populations, contexts and with a variable quality of reporting methods and results.

Finally, the availability of country-specific prevalence estimates of ASD over time will provide opportunities to undertake systematic assessments of the burden of disease and to promote evidence-based policy action [6, 60–62].

## Additional files

Additional file 1: PRISMA-P Checklist. (DOCX 32 kb)

Additional file 2: Key terms for PubMed/MEDLINE search. (DOCX 28 kb)

Additional file 3: Methodological Quality Checklist for Prevalence data. (DOCX 28 kb)

## Abbreviations

ASD: Autism spectrum disorder
DSM: *Diagnostic and Statistical Manual of Mental Disorders*
ICD: International Classification of Diseases
MOOSE: Meta-analysis Of Observational Studies in Epidemiology
PRISMA: Preferred Reporting Items for Systematic Reviews and Meta-Analyses
PRISMA-P: Preferred Reporting Items for Systematic Reviews and Meta-Analyses extension for Protocols
