# Supplementary material for: Prevalence and comorbidity of autism spectrum disorder in Spain: study protocol for a systematic review and meta-analysis of observational studies
Source: Syst Rev. 2019 Jun 14;8:141. doi: 10.1186/s13643-019-1061-1 (PMC6570970; doi:10.1186/s13643-019-1061-1)
Supplement: Supplementary file 2 — Key terms for PubMed/MEDLINE search. (DOCX 28 kb) [file 13643_2019_1061_MOESM2_ESM.docx]

**Additional file 2: Key terms for PubMed/MEDLINE search (.docx)**

Key terms for PubMed/MEDLINE search.

| Search | Query |
| --- | --- |
| #1 | (autism spectrum disorder*[All Fields] OR "autistic disorder"[MeSH Terms] OR autis*[All Fields] OR "autistic disorder"[All Fields] OR pervasive developmental disorder*[All Fields] OR "asperger syndrome"[MeSH Terms] OR Asperger*[All Fields]) |
| #2 | ("epidemiology"[Subheading] OR "epidemiology"[All Fields] OR "epidemiology"[MeSH Terms]) OR epidemiologic*[All Fields] OR cohort stud*[All Fields] OR cohort [All Fields] OR longitudinal stud*[All Fields] OR longitudinal [All Fields] OR "cross-sectional studies"[MeSH Terms] OR cross section*[All Fields] OR "prevalence"[MeSH Terms] OR prevalence*[All Fields]) |
| #3 | ("Spain"[MeSH Terms] OR spain*[Text Word]) OR espagne[All Fields] OR espana[All Fields] OR (spain[ad] OR espagne[ad] OR espana[ad]) OR osasunbidea[ad] OR osakidetza[ad] OR insalud[ad] OR sergas[ad] OR (catalunya[ad] OR catalonia[ad] OR catalogne[ad] OR cataluna[ad] OR catala[ad] OR (barcelon[ad] OR barcelona[ad] OR barcelone[ad] OR barcelones[ad] OR barceloneta[ad]) OR tarragona[ad] OR lleida[ad] OR lerida[ad] OR girona[ad] OR gerona[ad] OR sabadell[ad] OR hospitalet[ad] OR l'hospitalet[ad]) OR ((valencia[ad] OR valenciana[ad] OR valenciano[ad]) OR (castello[ad] OR castellon[ad]) OR alacant[ad] OR (alicant[ad] OR alicante[ad]) OR (murcia[ad] OR murcian[ad] OR murciana[ad] OR murciano[ad])) OR ((andalucia[ad] OR andaluciajunta[ad] OR andalusia[ad] OR andalusian[ad] OR andaluz[ad] OR andaluza[ad]) OR (sevill[ad] OR sevilla[ad] OR seville[ad]) OR (granada[ad] OR granade[ad]) OR huelva[ad] OR almeria[ad] OR cadiz[ad] OR jaen[ad] OR malaga[ad] OR (cordoba[ad] NOT (argentina[ad]) OR (extremadura[ad] OR caceres[ad] OR badajoz[ad] OR madrid[ad]) OR (castilla[ad] OR salamanca[ad] OR zamora[ad] OR valladolid[ad] OR segovia[ad] OR soria[ad] OR palencia[ad] OR avila[ad] OR burgos[ad]) OR (leon[ad] NOT (france[ad] OR clermont[ad] OR rennes[ad] OR lyon[ad] OR USA[ad] OR (mexic[ad] OR mexica[ad] OR mexican[ad] OR mexicana[ad] OR mexicano[ad] OR mexicanos[ad] OR mexico[ad]))) OR (galicia[ad] OR gallego[ad] OR compostela[ad] OR vigo[ad] OR coruna[ad] OR ferrol[ad] OR orense[ad] OR ourense[ad] OR pontevedra[ad]) OR (oviedo[ad] OR gijon[ad] OR (asturia[ad] OR asturiano[ad] OR asturias[ad])) OR ((cantabria[ad] OR cantabrico[ad] OR cantabro[ad]) OR santander[ad]) OR (vasco[ad] OR euskadi[ad] OR basque[ad] OR bilbao[ad] OR bilbo[ad] OR (donosti[ad] OR donostia[ad]) OR vizcaya[ad] OR guipuzcoa[ad] OR gipuzkoa[ad] OR alava[ad] OR araba[ad] OR vitoria[ad]) OR ((navarra[ad] OR navarro[ad]) OR pamplona[ad] OR iruna[ad] OR irunea[ad]) OR ((aragon[ad] OR aragones[ad]) OR zaragoza[ad] OR teruel[ad] OR huesca[ad]) OR (mancha[ad] OR ciudad real[ad] OR albacete[ad] OR cuenca[ad]) OR (toledo[ad] NOT (ohio[ad] OR us[ad] OR usa[ad] OR OH[ad])) OR (guadalajara[ad] NOT (mexic[ad] OR mexica[ad] OR mexican[ad] OR mexicana[ad] OR mexicano[ad] OR mexicanos[ad] OR OR mexico[ad])) OR ((balear[ad] OR balearen[ad] OR baleares[ad] OR balearic[ad] OR balears[ad] OR balearse[ad]) OR mallorca[ad] OR menorca[ad] OR ibiza[ad] OR eivissa[ad]) OR (palmas[ad] OR lanzarote[ad] OR (canaria[ad] OR canarian[ad] OR canarias[ad] OR canario[ad]) OR tenerife[ad]) |
| #4 | #1 AND #2 AND #3  No limits |
