# Supplementary material for: Prevalence and comorbidity of autism spectrum disorder in Spain: study protocol for a systematic review and meta-analysis of observational studies
Source: Syst Rev. 2019 Jun 14;8:141. doi: 10.1186/s13643-019-1061-1 (PMC6570970; doi:10.1186/s13643-019-1061-1)
Supplement: Supplementary file 3 — Methodological Quality Checklist for Prevalence data. (DOCX 28 kb) [file 13643_2019_1061_MOESM3_ESM.docx]

**Additional file 3: Methodological Quality Checklist for Prevalence data (.docx)**

Note: This scale has been adapted from the JBI Critical Appraisal Checklist for Studies Reporting Prevalence Data. The individual components listed below are summed to generate a total Methodological Quality score for each study. Total scores range from 0 to 10. For the total score grouping, studies were judged to be of low risk of bias (≥7 points), moderate risk of bias (4-6 points) and high risk of bias (<4 points).

1) **Was the sample representative of the target population?**

a) Yes**🟑**

b) No

c) Unclear/no description

d) Not applicable

2) **Were study participants recruited in an appropriate way?**

a) Yes**🟑**

b) No

c) Unclear/no description

d) Not applicable

3) **Was the sample size adequate?**

a) Yes**🟑**

b) No

c) Unclear/no description

d) Not applicable

4) **Were the study subjects and the setting described in detail?**

a) Yes**🟑**

b) No

c) Unclear/no description

d) Not applicable

5) **Was the data analysis conducted with sufficient coverage of the identified sample?**

a) Yes**🟑**

b) No

c) Unclear/no description

d) Not applicable

6) **Were the objective, standard criteria used for the measurement of the condition?**

a) Yes**🟑**

b) No

c) Unclear/no description

d) Not applicable

7) **Was the condition measured reliably?**

a) Yes**🟑**

b) No

c) Unclear/no description

d) Not applicable

8) **Was there an appropriate reporting of statistical analysis?**

a) Yes**🟑**

b) No

c) Unclear/no description

d) Not applicable

9) **Are all important confounding factors, subgroups, or potential differences identified and accounted for?**

a) Yes**🟑**

b) No

c) Unclear/no description

d) Not applicable

10) **Were subpopulations identified using objective criteria?**

a) Yes**🟑**

b) No

c) Unclear/no description

d) Not applicable
